# The velocity of temporalis muscle wasting in cerebral metastasis is prognostic for poor survival

**DOI:** 10.3389/fonc.2025.1482705

**Published:** 2025-02-28

**Authors:** Artem Rafaelian, Sae-Yeon Won, Bedjan Behmanesh, Daniel Cantré, Joshua D. Bernstock, Thomas M. Freiman, Jakob Seidlitz, Peter Baumgarten, Nazife Dinc, Juergen Konczalla, Florian Gessler, Daniel Dubinski

**Affiliations:** ^1^ Department of Neurosurgery, Rostock University Medical Center, Rostock, Germany; ^2^ Institute of Diagnostic and Interventional Radiology, Pediatric Radiology and Neuroradiology, University Medicine Rostock, Rostock, Germany; ^3^ Department of Neurosurgery, Brigham and Women’s Hospital, Harvard Medical School, Boston, MA, United States; ^4^ Lifespan Brain Institute, The Children’s Hospital of Philadelphia and Penn Medicine, Philadelphia, PA, United States; ^5^ Department of Psychiatry, University of Pennsylvania, Philadelphia, PA, United States; ^6^ Institute for Translational Medicine and Therapeutics, University of Pennsylvania, Philadelphia, PA, United States; ^7^ Department of Neurosurgery, University Hospital Augsburg, Augsburg, Germany; ^8^ Department of Neurosurgery, University Hospital, Schiller University Jena, Jena, Germany; ^9^ Department of Neurosurgery, Goethe-University Hospital, Frankfurt am Main, Germany

**Keywords:** sarcopenia, cerebral metastases, survival analysis, frailty, temporal muscle

## Abstract

**Purpose:**

Recently, the temporalis muscle thickness on cranial CT scan was proposed as a surrogate marker for patients’ baseline frailty that correlates with outcome in primary and metastatic brain tumor patients. In this study, we investigated whether the velocity of temporalis muscle atrophy (TMA) affects the outcome of patients with cerebral metastases.

**Methods:**

We analyzed radiological and clinical data sets of 96 patients who received craniotomy for cerebral metastasis resection in our institution. We then correlated the radiological data with clinical course and outcome after stratification for the velocity of temporalis muscle atrophy.

**Results:**

The median velocity of TMA was 0.0016 mm/day. In patients with a slow TMA rate, the median overall survival was significantly longer than in patients with a fast TMA rate (37.7 months versus 22.9, *p* = 0.0007). Furthermore, patients with slow TMA had longer progression-free survival postoperatively (7.6 versus 4.38 months, p <0.0001). The overall survival postoperatively (OS-PO) was also significantly longer in patients with slow TMA (8.9 months versus 5.1, p=0002).

**Conclusion:**

Based on this study, the velocity of temporalis muscle atrophy may represent an objective and dynamic index with potential for survival prognostication for patients with cerebral metastases.

## Introduction

With advances in screening, imaging, and therapy, survival and the quality of life has improved annually in cancer patients. However, cerebral metastasis remains a major source of persistent neurological deficits, disability, and mortality, and 20% to 40% of all solid tumors metastasize to the brain at some point in time, changing the approach to cancer treatment and significantly limiting the ability to participate in clinical trials (1–3). The decision on further adjuvant treatment and its intensity largely depends on the prognosis of the underlying disease (4).

Currently, several approaches have been developed for prognostication of cancer progression both on the basis of mathematical analysis and on the basis of artificial intelligence (5, 6). Widely adopted scales in the assessment of patients’ frailty and therefore suitability of neo- and adjuvant therapy are the Karnofsky Performance Status (KPS) and the Eastern Cooperative Oncology Group (ECOG) Performance status. One of the disadvantages however is the subjective nature of these scales and the potential for biased reporting. On the other hand, scientific interest in the objective assessment of patient’s physiological reserve is growing and patient’s frailty estimation via the surrogate temporalis muscle thickness (TMT) measurement has gained increasing interest due to the ability to measure via non-invasive head imaging (7).

The pathobiology underlying sarcopenia and frailty in individuals with brain metastases is complex, with underlying factors such as chemo-radiation and immune therapy-induced hypodynamia, malnutrition and/or dietary factors, and tumor-induced inflammation fostering a pro-catabolic state characterized by heightened protein degradation relative to synthesis—all culminating in skeletal muscle tissue loss and autophagy. Klicken oder tippen Sie hier, um Text einzugeben (8, 9).Klicken oder tippen Sie hier, um Text einzugeben. On the other hand, the TMT measurement on MRI or CT scan is easy to obtain and recent studies showed a positive correlation between the thickness of temporalis muscle and patients’ outcome in neuro-trauma and oncological diseases. Klicken oder tippen Sie hier, um Text einzugeben (10–16).

In this study, we consistently moved away from the static TMT measurement at the beginning of the disease and investigated the influence of frailty dynamics on the clinical course and outcome. Therefore, the aim of this study was primarily to investigate the dynamics of frailty, measured by TMT, and its potential for predicting clinical outcomes in cancer patients with brain metastases. We chose temporalis muscle data because this muscle is almost always clearly depicted on MRI scans, which are used both preoperatively and for regular follow-up examinations. In particular, we aim to evaluate whether changes in TMT over time can serve as a more reliable marker of frailty compared with static preoperative measurements alone, as described by other authors. This dynamic approach is expected to more accurately predict clinical outcomes and response to treatment, given the variable nature of frailty in cancer patients. The dynamics of frailty, especially as reflected in the rate of change in TMT, may provide insights into how cancer progression, treatment interventions, and patient responses lead to changes in the patient’s physiological reserve.

## Materials and methods

### Patient selection

All patients admitted to the neurosurgical department at the University Hospital Rostock between 2018 and 2022 who underwent surgical resection for parenchymal brain metastases were included. Indication for craniotomy were neurological deficit, metastasis size unsuitable for radiotherapy, and/or tumor size >3 cm in diameter. The indication for surgical treatment was confirmed in advance at an interdisciplinary tumor conference for all cases. Exclusion methods were missing MR imaging before and/or after surgical intervention in the radiology facility of our university. A total of 96 patients were included in the study. Patient characteristics and medical data were collected via the institution’s electronic database. For this retrospective analysis, ethical approval was obtained from the Ethics Committee of the University Medicine Rostock, Germany (Identification number: A 2021-0112); as a non-interventional, retrospective study, patient consent was waived. The collected data included age at surgical examination, gender, BMI, the amount of dexamethasone received 2 weeks before and after surgery, the date of the first oncological diagnosis, histological and mutational tumor characteristics, the presence of metastasis in other organs, assessment of cerebral metastasis (size, quantity, cerebral edema) concomitant diseases, median TMT before surgery and in the last follow-up, perioperative complications, KPS prior to surgery, the presence of adjuvant and neoadjuvant treatment, the Graded Prognostic Assessment index, and the date of the last inspection, contact, or death. Due to the heterogeneity of the underlying oncological disease and the subject-specific definition of progression-free survival, we did not include the progression-free survival in our analysis and restricted this study to patients’ overall survival, progression-free survival, and overall survival postoperatively (OS, PFS-PO, and OS-PO, respectively).

### Assessment of temporalis muscle thickness

**Figure 1:**
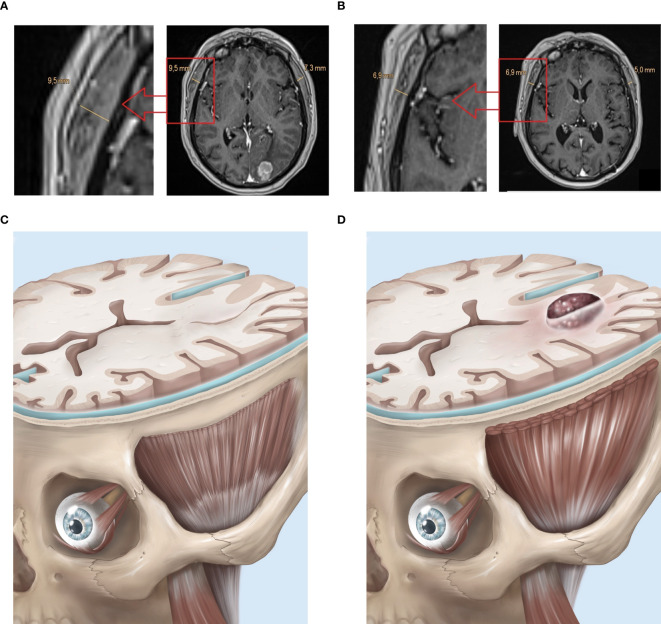
Exemplary case depicting temporalis muscle atrophy (TMA) in a case of NSCLC (EGFR, ALK: neg.) with singular cerebral metastasis, treated with metastasis resection and combined immune- and radiation therapy. TMT-Measurements assessed in axial reconstructions of contrast enhanced T1-weighted MRI sequences **(A)** in the preoperative staging MRI (resulting mean TMT_1_ = (9.5 mm + 7.3 mm)/2 = 8.4 mm) and **(B)** in the final follow-up MRI (TMT_2_ = 5.95 mm) after combined treatment with good response. The time interval between these MRIs was 421 days. The rate of TMA was V = (8.4–5.95)/421 = 0.00581 mm/day. **(C)** Illustrative model with occipital lobe metastasis at the beginning of the treatment with healthy TMT. D: Same model with TMA after treatment.

TMT measurements were performed on contrast-enhanced T1-weighted MR images (T1-mprage with 1-mm isotropic voxel size), in standardized axial reconstructions, by a reader blinded to the outcome. For each patient, we performed these measurements in the preoperative staging MRI examination (TMT_1_), as well as follow-up MRI examinations until the last examination, last before lost to follow-up, or patients’ decease (TMT_2_). TMT was measured perpendicular to the long axis of the temporalis muscle at the level of the Sylvian fissure (anteroposterior landmark) and the roof of the orbit (craniocaudal landmark). If the axial plane was tilted from the desired plane during image registration, the image was aligned to the required plane using the 3D-MPR mode. The measurements were performed separately for both sides, and then the arithmetic mean was calculated, resulting in a mean TMT value for each patient and timepoint. If the craniotomy involved the temporal muscle (e.g., temporal approach or pterional approach), then the thickness of the temporal muscle was calculated both pre- and postoperatively on the “healthy” side only. The time interval between examination timepoints was also determined in numbers of days (n). Thus, the rate of temporalis muscle atrophy (TMA) was obtained according to the formula V = (mean TMT_1_ − mean TMT_2_)[mm]/n [day]. Preoperative and postoperative images and subsequent MRI scans were analyzed using PACS software JiveX^®^ v5.2 (VISUS Technology Transfer GmbH, Bochum, Germany). Image analysis was performed independently by two neurosurgeons who was blinded to patients’ medical data, and the mean of both measurements was calculated. A representative analysis is displayed in **Figure 1**.

### Statistics

We calculated overall survival (OS) using days from the first diagnosis to the death date and OS postoperative (OS-PO) using days from the operation to the death date. Kaplan–Meier survival curve analysis and the log-rank test were conducted to obtain the median OS of the groups. Univariate and multivariate analyses were performed using a Cox proportional regression model. To assess the impact of the variables, odds ratios (ORs) with 95% confidence intervals (CIs) were calculated. Results with a p-value of ≤0.05 were considered to be statistically relevant. All statistical analyses were conducted using the GraphPad Prism 10 (GraphPad Software, California, USA). For patient characteristics, descriptive statistics were used.

## Results

### Cohort characteristics

The study group consisted of 121 patients; in 25 patients (20.6%), medical datasets and/or follow-up data were incomplete, and therefore 96 patients remained in the study. The average age was 64 years old (IQR: 56.8–71.3), and 55 (57.3%) of the patients were men. In 34 (35.4%) cases, symptomatic cerebral metastases lead to the first oncological diagnosis. The median TMT at admission was 5.95 mm (IQR: 5.1–7.0). The median total dexamethasone doses that patients were administered (total count for 14 days) pre-OP and post-OP were 36 mg (IQR: 0–72) and 60 mg (IQR: 19.5–93.75), respectively. It is noteworthy that eight (8.3%) patients had an increase in muscle mass. Patient characteristics are shown in **Table 1**.

**Table 1:** Demographics.

Gender, n (%)	Male, 55 (57.3%)
	Female, 41 (42.7)
Mean age at diagnosis, years (IQR)	64 (56.8–71.3)
Initial diagnosis through cerebral metastasis, n (%)	34 (35.4%)
Median of TMT, mm (IQR)	5.95 (5.1-7.0)
BMI, kg/m^2^ (IQR)	26.3 (24.2-29.3)
Median of the total dexamethasone dosage (total count for 14 days), mg (IQR)	
Pre-OP	36 (0-72)
Post-OP	60 (19.5-93.75)
Median size of cerebral metastasis, cm^3^ (IQR)	12.58 (6.76-22.82)
Median size of edema, cm^3^ (IQR)	65.99 (33.51-122.45)
Number of brain metastasis, n (%)	
1	71 (73.9%)
>1	25 (26.1%)
Primary cancer type, *n* (%)	
NSCLC	46 (47.9%)
RCC	10 (10.4%)
SCLC	10 (10.4%)
Melanoma	8 (8.3%)
Breast cancer	7 (7.3%)
CRC	6 (6.3%)
Ovarian cancer	3 (3.1%)
Others	6 (6.3%)
Complications, n (%)	
Postoperative infection	16 (13.2%)
Postoperative hemorrhage	2 (1.65%)
Adjuvant therapy, n (%)	
Stereotactic radiosurgery	15 (15.6%)
Radiation therapy	64 (66.6%)
Without treatment	17 (17.7%)
GPA n (%)	
4	2 (2.1%)
3.5-3	21 (21.8%)
2-2.5	37 (38.5%)
1-1.5	28 (29.2%)
0-0.5	3 (3.1%)
Unknown	5 (5.2%)
KPS, n (%)	
100-80	32 (33.3%)
<80	64 (66.7%)
Median velocity of temporalis muscle atrophy, mm/day (IQR)	0.001653 (0.000387-0.004117)
Median of OS. month (IQR)	26.6 (9.33-50.23)
Median of OS-PO. month (IQR)	6.2 (2.6-14.3)
Median of PFS-PO. month (IQR)	5 (2.3-13.16)

BMI, body mass index; IQR, interquartile range; NSCLC, non-small cell lung cancer; SCLC, small cell lung cancer; CRC, colorectal carcinoma; RCC, renal cell carcinoma; GPA, Graded Prognostic Assessment; KPS, Karnofsky performance status; TMT, temporalis muscle thickness; OS, overall survival; OS-PO, overall survival postoperative; PFS-PO, progression-free survival overall survival.

We used the median rate of TMA (0.001653 mm/day) to stratify the cohort. The cohort (n=96) was divided into groups with high and low rates of TMT atrophy based on this median value. A comparative analysis of the data of the two groups was carried out. We would like to point out that both groups in our study were quite homogeneous in terms of patient gender, whether the diagnosis of brain metastasis was the first oncologic diagnosis, and interestingly, both groups showed an almost equal number of patients who refused further adjuvant treatment.

The univariate analysis showed no significant association between patients’ sex (*p* = 0.84), BMI (*p* = 0.4872), or median TMT at admission (*p* = 0.77) and the velocity of temporalis muscle atrophy. Furthermore, there was no significant difference between the KPS (*p* =0.0668) or GPA (*p* = 0.17-0.99) and the velocity of temporalis muscle atrophy. Such an indicator as the age of patients showed a difference in the two groups, but in our sample of patients, it was statistically insignificant (63 versus 59.5; *p* = 0.15). The detailed results are presented in **Table 2**.

**Table 2:** Univariate analysis of juxtaposed characteristics according to the rate of TMT muscle atrophy.

Patient characteristic	Fast TMT atrophy >0.001653 mm/day	Slow TMT atrophy <0.001653 mm/day	Univariate		
n = 96	n=48	n=48	OR	95% CI	*p*-value
Sex male, n (%)	28 (58.3)	27 (56.3)	1.0889	0.1817–0.2234	0.8386
Age, median (IQR)	63 (56.75-70.25)	59.5 (54-68.25)	–	1.166-7.500	0.1501
Initial diagnosis through cerebral metastasis, n (%)	19 (39.6%)	15 (31.3%)	1.4414	0.1601–0.2434	0.6827
Median of TMT, mm (IQR)	6.1 (5.23-7.26)	6.08 (5.24-7.18)	–	0.5341–0.7154	0.7740
BMI, kg/m^2^ (IQR)	25.6 (22.4-30.2)	25.3 (21.7-28.2)	–	1.468-3.059	0.4872
Median of the total dexamethasone dosage (total count for 14 days), mg (IQR)					
Pre-OP, mg (IQR)	24 (0-66)	36 (0-73)	–	19.43–22.34	0.8900
Post-OP, mg (IQR)	60 (28-108.5)	51.5 (25.5-91.75)	–	26.47–18.17	0.7131
Median size of cerebral metastasis, cm^3^ (IQR)	14.4 (8.69-29.48)	9.7 (5.26-18.33)	–	0.1383–14.17	0.0457*
Median size of edema, cm^3^ (IQR)	67.25 (39.94-158.54)	62.56 (26.32-113.87)	–	1494-4426	0.3279
Number of brain metastasis, n (%)					
1	32 (66.7%)	39 (81.2%)	0.4615	0.03142–0.3231	0.1057
>1	16 (33.3%)	9 (18.8%)	2.1667		
Primary cancer type, *n* (%)					
NSCLC	22 (45.8%)	24 (50%)	0.8462	0.1628–0.2461	0.6866
RCC	5 (10.4%)	5 (10.5%)	1	0.1251–0.1251	>0.9999
SCLC	7 (14.8%)	3 (6.2%)	2.561	0.2073–0.04062	0.1851
Melanoma	5 (10.4%)	3 (6.2%)	1.7442	0.1545–0.07121	0.4654
Breast cancer	3 (6.2%)	4 (8.4%)	0.7333	0.08557–0.1272	0.6983
CRC	3 (6.2%)	3 (6.2%)	1	0.09914–0.09914	>0.9999
Others	3 (6.2%)	6 (12.5%)	0.4667	0.1376–0.01257	0.1016
Complications, n (%)					
Postoperative infection	9 (18.6%)	7 (14.8%)	1.3516	0.1941–0.1107	0.5885
Postoperative hemorrhage	1 (2.1%)	1 (2.1%)	1	0.05850–0.05850	>0.9999
Adjuvant therapy, n (%)					
Stereotactic radiosurgery	7 (14.8%)	8 (16.6%)	0.8537	0.1278–0.1695	0.7814
Radiation therapy	33 (68.6%)	31 (64.6%)	1.2065	0.1512–0.2346	0.6690
without treatment	8 (16.6%)	9 (18.8%)	0.8667	0.1355–0.1771	0.7919
GPA, n (%)					
4	1 (2.1%)	1 (2.1%)	1	0.05850–0.05850	>0.9999
3.5-3	10 (20.8%)	11 (22.9%)	0.8852	0.1484–0.1901	0.8075
2-2.5	21 (43.8%)	16 (33.3%)	1.5556	0.3024–0.09403	0.2994
1-1.5	13 (27%)	15 (31.2%)	0.8171	0.1443–0.2276	0.6574
0-0.5	2 (4.2%)	1 (2.1%)	2.0435	0.09197–0.05030	0.5623
Unknown	1 (2.1%)	4 (8.4%)	0.234	0.02760–0.1526	0.1717
KPS					
100-80	21 (43.8%)	11 (22.9%)	2.6162	0.01325–0.3883	0.0668
<80	27 (56.2%)	37 (77.1%)	0.3822		
Median of OS. month (IQR)	22.9 (11.5-32.3)	37.7 (25.8-57.5)	–	0.2624–0.7562	0.0007***
Median of OS-PO. month (IQR)	5.1 (2.6-14.6)	8.9 (3.88-19.3)	–	0.3050–0.8685	0.0002***
Median of PFS-PO. month (IQR)	4.38 (2.3-11.9)	7.6 (4.3-23.1)	–	0.1731–0.4236	<0.0001****

BMI, body mass index; OR, odds ratio; IQR, interquartile range; NSCLC, non-small cell lung cancer; SCLC, small cell lung cancer; CRC, colorectal carcinoma; RCC, renal cell carcinoma; GPA, graded prognostic assessment; KPS, Karnofsky performance status; TMT, temporalis muscle thickness; OS, overall survival; OS-PO, overall survival postoperative; PFS-PO, progression-free survival overall survival. * p< 0.10 (weak significance); ** p< 0.05 (moderate significance); *** p< 0.01 (high significance); **** p< 0.0001 (very high significance).

**Figure 2:**
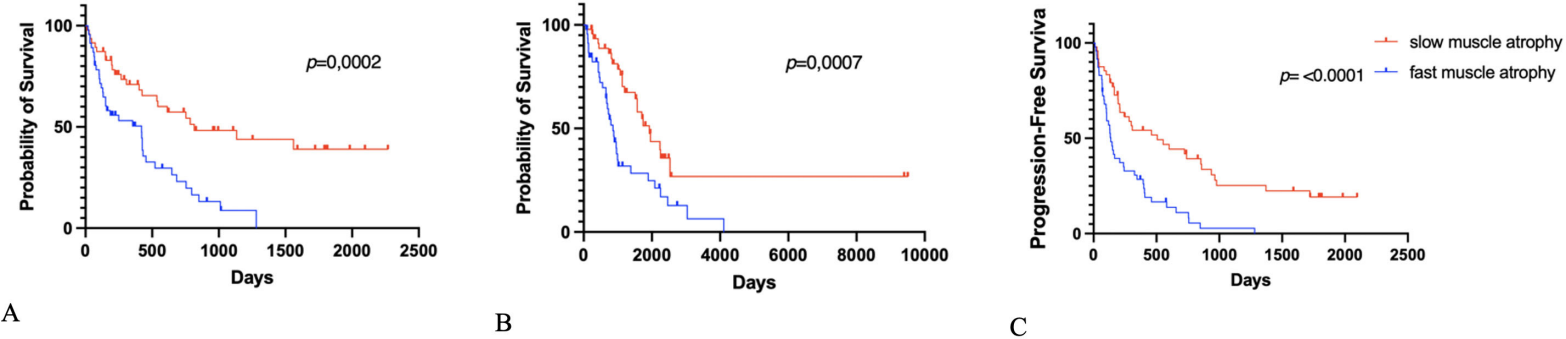
Kaplan–Meier survival graphs of **(A)** overall survival, **(B)** overall survival postoperatively (OS-PO), and **(C)** progression-free survival postoperatively (PFS-PO) stratified according to fast und slow TMT atrophy.

**Figure 3:**
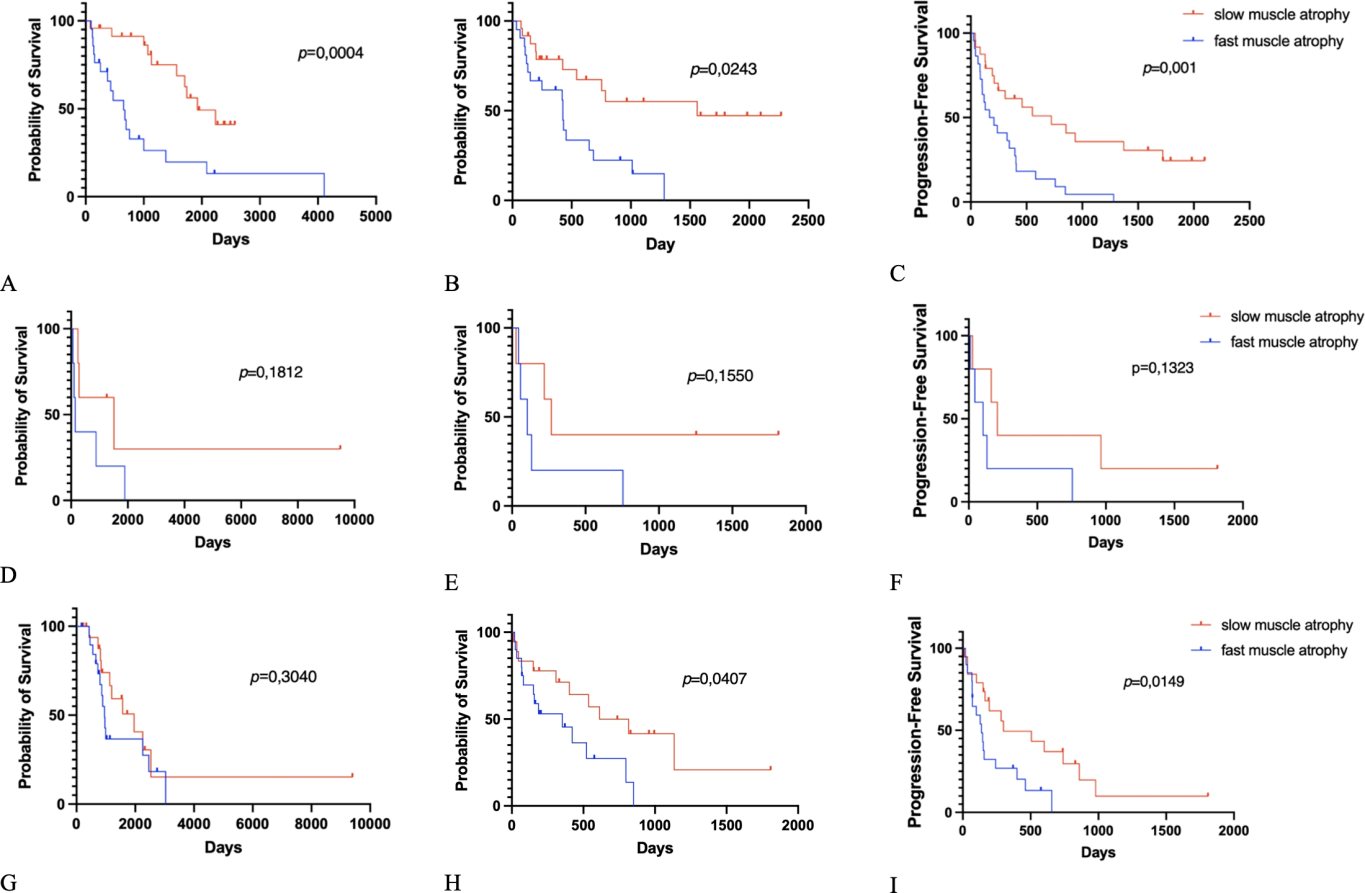
Kaplan–Meier survival graphs: **(A)** Overall survival (OS), **(B)** OS-postoperative (OS-PO), **(C)** progression-free survival postoperative (PFS-PO) for fast and slow muscle degeneration and non-small cell lung cancer. **(D)** OS, **(E)** OS-PO, **(F)** PFS-PO for fast and slow muscle degeneration and renal cell carcinoma. **(G)** OS, **(H)** OS-PO, **(I)** PFS-PO for fast and slow muscle degeneration and all other tumors.

The Kaplan–Meier overall survival, progression-free survival, and overall survival postoperatively each subgroup are presented in **Figures 2**, **3**.

## Discussion

A significant association between the velocity of TMA and the median size of cerebral metastasis was observed (median size of cerebral metastasis of 14.4 cm^3^ (IQR: 8.69–29.48) in the fast TMT atrophy cohort vs. 9.7 cm^3^ (IQR: 5.26–18.33) in the Slow TMT atrophy cohort; p = 0.0457).

In the group with a slow TMT atrophy rate, median OS was significantly longer than in the group with a fast TMT atrophy rate (37.7 (IQR: 25.8-57.5) versus 22.9 (IQR: 11.5-32.3) months, *p* = 0.0007). The group with a slow TMT atrophy rate had longer progression-free survival postoperative (PFS-PO) (7.6 (IQR: 4.3-23.1) versus 4.38 (IQR: 2.3-11.9) months, *p <*0.0001). In the group with a slow TMT atrophy rate, median OS-PO was significant longer than in the group with fast TMT atrophy (8.9 (IQR: 3.88-19.3) versus 5.1 (2.6-14.6) months, *p* = 0.0002).

In this study, we hypothesized that time-dependent TMA could be used as a dynamic index to objectify patients’ frailty throw-out adjuvant treatment. We used the median rate of TMA (0.001653 mm/day) for cohort stratification. The survival analysis showed a statistically highly significant difference in all three key oncologic indicators OS, OS-PO, and PFS-PO (p=0.0007; p=0.0002; p<0.0001). Unfortunately, we have no data on the change in BMI in patients during treatment, as the study is retrospective and not all patients had this index recorded at control MRI. We recognize that this index would be interesting for comparison. However, it did not show a statistically significant difference in the fast and slow temporal muscle atrophy groups at the onset of the disease.

Surprisingly, our analysis revealed no statistically significant difference in patient age (p=0.150) and sex distribution (p=0.838), variables recognized in various studies as prognostic factors. This underscores the importance of incorporating dynamic indicators (such as fitness level, weight loss, or quality of nutrition) that may evolve during the course of treatment (17). The question of finding direct correlations between a patient’s “biological age” and the presence of objective biomarkers is becoming increasingly common (18).

Sarcopenia, meanwhile, finds itself trapped in a cycle where aggressive adjuvant and neoadjuvant therapies contribute to its development, and its progression, in turn, leads to poorer treatment outcomes. As highlighted in Bozzetti’s article, various mechanisms of sarcopenia pathogenesis are discussed (19). There are various studies linking sarcopenia to muscle protein catabolism, which intensifies as tumor growth progresses (20, 21). Some side effects of chemotherapy, such as fatigue, loss of appetite, nausea, vomiting, and diarrhea, can negatively affect food intake and physical activity (22). Chemotherapy may suppress anabolic processes through the IGF-1/PI3K/Akt/mTOR pathway, which also contributes to muscle loss (23). Chemotherapy and radiation can also reduce microvessels in muscle through anti-angiogenesis and cause localized muscle tissue damage, inflammation, and fibrosis, reducing its functionality (24).

While numerous publications have previously elucidated the deleterious impact of sarcopenia on cancer prognosis, our objective is to underscore the rate of sarcopenia index development within this context. Panje et al.’s (25) research indicates that sarcopenia’s onset during treatment may not only prognosticate outcomes but also elevate the risk of severe complications during adjuvant radiochemotherapy. Furthermore, the noteworthy study by Jang et al. suggests that diverse neoadjuvant therapies for breast cancer patients may variably influence sarcopenia progression, reinforcing the pertinence of this field and the necessity for further prospective investigations. Consequently, interventions aimed at preserving muscle mass, such as exercise regimens and resistance training, alongside nutritional support throughout various disease stages—including adjuvant, surgical, and neoadjuvant treatments—may prove pivotal in enhancing disease prognosis (26, 27).

Given the heterogeneous treatment response and survival outcomes among patients with cerebral metastases, numerous studies have sought to forecast prognosis through classification scoring systems (4). Among these is the Graded Prognostic Assessment (GPA), introduced by Sperduto et al., which stands out as a widely utilized metric in the field. Specifically designed for disease-specific evaluation, the GPA scale furnishes a prognostic index adapted to the diverse manifestations of brain metastasis across various primary cancers (28). However, a significant limitation of this method lies in its predominantly subjective nature, thus prompting consideration for the inclusion of more objective parameters of frailty, such as TMT atrophy (29).

Our colleagues have previously demonstrated a significant correlation between overall life expectancy in malignant brain tumors and TMT. Subsequently, Furtner et al. conducted a comprehensive retrospective study involving 755 glioblastoma patients, stratifying them into two groups based on TMT measurements. Sarcopenia risk was defined as TMT values ≤6.3 mm for men and ≤5.2 mm for women. Results revealed that patients exhibiting sarcopenia at baseline displayed a markedly elevated risk for disease progression and mortality compared with those with normal muscle status (30).

While our analysis underscores the importance of TMT atrophy velocity throughout the course of oncological disease and treatment in a substantial cohort of brain metastasis patients, our study is subject to certain constraints. The retrospective design of our TMT analysis precluded the evaluation of anatomical-functional correlations. Furthermore, given the observational nature of our study, potential confounding variables (such as comorbidities, refusal of treatment and therapy, accidents) and uncontrolled statistical errors cannot be excluded. Thus, future prospective randomized investigations involving larger cohorts are warranted to confirm our findings. Unfortunately due to the limited number of patients, multivariate analysis did not show significant differences in this regard; we will continue our study with a further prospective phase.

## Conclusion

Based on our analysis, we propose monitoring the TMA dynamics longitudinally to facilitate the identification of suitable candidates for intensive therapeutic interventions or enrollment in clinical trials.

## Data Availability

The raw data supporting the conclusions of this article will be made available by the authors, without undue reservation.
